# Role of human plasma metabolites in prediabetes and type 2 diabetes from the IMI-DIRECT study

**DOI:** 10.1007/s00125-024-06282-6

**Published:** 2024-09-30

**Authors:** Sapna Sharma, Qiuling Dong, Mark Haid, Jonathan Adam, Roberto Bizzotto, Juan J. Fernandez-Tajes, Angus G. Jones, Andrea Tura, Anna Artati, Cornelia Prehn, Gabi Kastenmüller, Robert W. Koivula, Paul W. Franks, Mark Walker, Ian M. Forgie, Giuseppe Giordano, Imre Pavo, Hartmut Ruetten, Manolis Dermitzakis, Mark I. McCarthy, Oluf Pedersen, Jochen M. Schwenk, Konstantinos D. Tsirigos, Federico De Masi, Soren Brunak, Ana Viñuela, Andrea Mari, Timothy J. McDonald, Tarja Kokkola, Jerzy Adamski, Ewan R. Pearson, Harald Grallert

**Affiliations:** 1 Research Unit of Molecular Epidemiology, Institute of Epidemiology, German Research Center for Environmental Health, Helmholtz Zentrum München, Neuherberg, Germany; 2 Faculty of Medicine, Ludwig-Maximilians-University München, Munich, Germany; 3 Metabolomics and Proteomics Core, German Research Center for Environmental Health, Helmholtz Zentrum München, Neuherberg, Germany; 4 German Center for Diabetes Research (DZD), München Neuherberg, Germany; 5 Institute of Neuroscience, National Research Council, Padova, Italy; 6 Wellcome Trust Centre for Human Genetics, University of Oxford, Oxford, UK; 7 Department of Clinical and Biomedical Sciences, University of Exeter College of Medicine & Health, Exeter, UK; 8 Institute of Computational Biology, Helmholtz Zentrum München, Munich, Germany; 9 Oxford Centre for Diabetes, Endocrinology and Metabolism, University of Oxford, Oxford, UK; 10 Department of Clinical Science, Genetic and Molecular Epidemiology, Lund University Diabetes Centre, Malmö, Sweden; 11 Translational and Clinical Research Institute, Faculty of Medical Sciences, University of Newcastle, Newcastle upon Tyne, UK; 12 Population Health and Genomics, Ninewells Hospital and Medical School, University of Dundee, Dundee, UK; 13 Department of Clinical Science, Genetic and Molecular Epidemiology, Lund University Diabetes Centre, Malmö, Sweden; 14 Eli Lilly Regional Operations GmbH, Vienna, Austria; 15 Sanofi Partnering, Sanofi-Aventis Deutschland GmbH, Frankfurt am Main, Germany; 16 Department of Genetic Medicine and Development, University of Geneva Medical School, Geneva, Switzerland; 17 Institute for Genetics and Genomics in Geneva (iGE3), University of Geneva, Geneva, Switzerland; 18 Swiss Institute of Bioinformatics, Geneva, Switzerland; 19 Center for Clinical Metabolic Research, Herlev and Gentofte University Hospital, Copenhagen, Denmark; 20 Novo Nordisk Foundation Center for Basic Metabolic Research, Faculty of Health and Medical Sciences, University of Copenhagen, Copenhagen, Denmark; 21 Science for Life Laboratory, School of Biotechnology, KTH - Royal Institute of Technology, Solna, Sweden; 22 Department of Health Technology, Technical University of Denmark, Kongens Lyngby, Denmark; 23 Department of Health Technology, Technical University of Denmark, Kongens Lyngby, Denmark; 24 Biosciences Institute, Faculty of Medical Sciences, University of Newcastle, Newcastle upon Tyne, UK; 25 Blood Sciences, Royal Devon and Exeter NHS Foundation Trust, Exeter, UK; 26 Internal Medicine, Institute of Clinical Medicine, University of Eastern Finland, Kuopio, Finland; 27 Department of Biochemistry, Yong Loo Lin School of Medicine, National University of Singapore, Singapore, Singapore; 28 Institute of Experimental Genetics, German Research Center for Environmental Health, Helmholtz Zentrum München, Neuherberg, Germany; 29 Institute of Biochemistry, Faculty of Medicine, University of Ljubljana, Ljubljana, Slovenia

**Keywords:** Causality, Glycaemic traits, HbA_1c_, IMI-DIRECT, Mediation, Metabolomics, *N*-lactoylaminoacids, Patient stratification, Targeted metabolomics, Type 2 diabetes, Untargeted metabolomics

## Abstract

**Aims/hypothesis:**

Type 2 diabetes is a chronic condition that is caused by hyperglycaemia. Our aim was to characterise the metabolomics to find their association with the glycaemic spectrum and find a causal relationship between metabolites and type 2 diabetes.

**Methods:**

As part of the Innovative Medicines Initiative - Diabetes Research on Patient Stratification (IMI-DIRECT) consortium, 3000 plasma samples were measured with the Biocrates Absolute*IDQ* p150 Kit and Metabolon analytics. A total of 911 metabolites (132 targeted metabolomics, 779 untargeted metabolomics) passed the quality control. Multivariable linear and logistic regression analysis estimates were calculated from the concentration/peak areas of each metabolite as an explanatory variable and the glycaemic status as a dependent variable. This analysis was adjusted for age, sex, BMI, study centre in the basic model, and additionally for alcohol, smoking, BP, fasting HDL-cholesterol and fasting triacylglycerol in the full model. Statistical significance was Bonferroni corrected throughout. Beyond associations, we investigated the mediation effect and causal effects for which causal mediation test and two-sample Mendelian randomisation (2SMR) methods were used, respectively.

**Results:**

In the targeted metabolomics, we observed four (15), 34 (99) and 50 (108) metabolites (number of metabolites observed in untargeted metabolomics appear in parentheses) that were significantly different when comparing normal glucose regulation vs impaired glucose regulation/prediabetes, normal glucose regulation vs type 2 diabetes, and impaired glucose regulation vs type 2 diabetes, respectively. Significant metabolites were mainly branched-chain amino acids (BCAAs), with some derivatised BCAAs, lipids, xenobiotics and a few unknowns. Metabolites such as lysophosphatidylcholine a C17:0, sum of hexoses, amino acids from BCAA metabolism (including leucine, isoleucine, valine, *N-*lactoylvaline, *N*-lactoylleucine and formiminoglutamate) and lactate, as well as an unknown metabolite (X-24295), were associated with HbA_1c_ progression rate and were significant mediators of type 2 diabetes from baseline to 18 and 48 months of follow-up. 2SMR was used to estimate the causal effect of an exposure on an outcome using summary statistics from UK Biobank genome-wide association studies. We found that type 2 diabetes had a causal effect on the levels of three metabolites (hexose, glutamate and caproate [fatty acid (FA) 6:0]), whereas lipids such as specific phosphatidylcholines (PCs) (namely PC aa C36:2, PC aa C36:5, PC ae C36:3 and PC ae C34:3) as well as the two *n*-3 fatty acids stearidonate (18:4n3) and docosapentaenoate (22:5n3) potentially had a causal role in the development of type 2 diabetes.

**Conclusions/interpretation:**

Our findings identify known BCAAs and lipids, along with novel *N*-lactoyl-amino acid metabolites, significantly associated with prediabetes and diabetes, that mediate the effect of diabetes from baseline to follow-up (18 and 48 months). Causal inference using genetic variants shows the role of lipid metabolism and *n*-3 fatty acids as being causal for metabolite-to-type 2 diabetes whereas the sum of hexoses is causal for type 2 diabetes-to-metabolite. Identified metabolite markers are useful for stratifying individuals based on their risk progression and should enable targeted interventions.

**Graphical Abstract:**

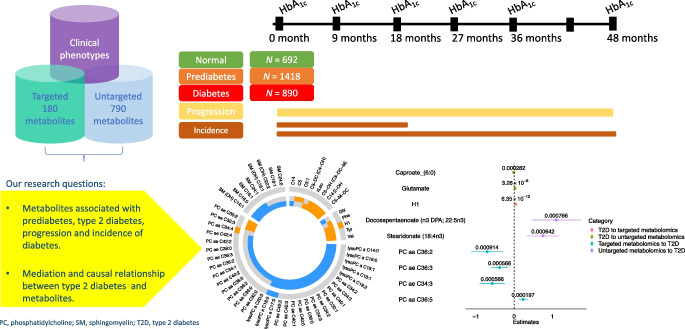

**Supplementary Information:**

The online version contains peer-reviewed but unedited supplementary material available at 10.1007/s00125-024-06282-6.


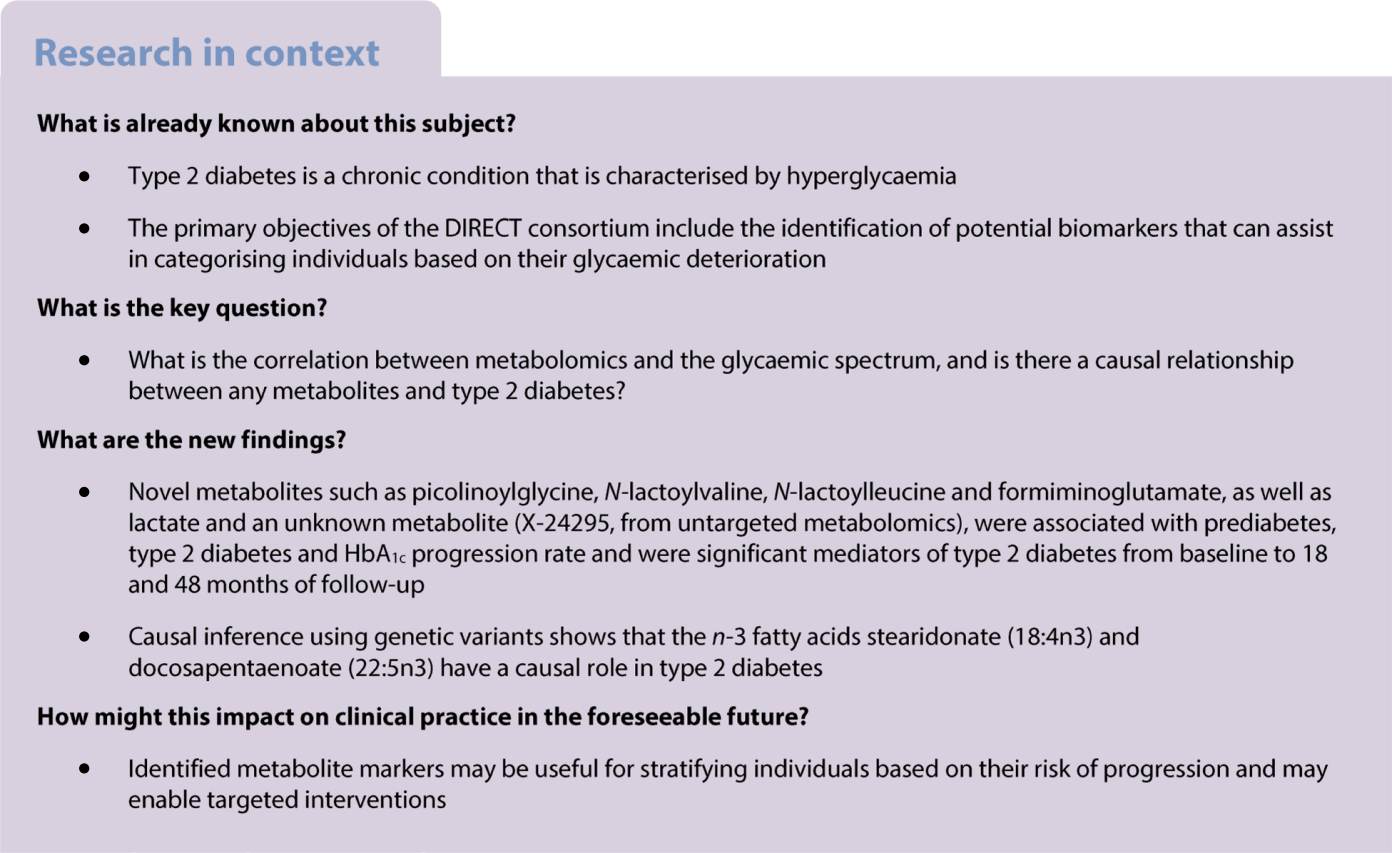


## Introduction

Type 2 diabetes is a complex and common metabolic disorder, resulting from the body’s ineffective use of insulin. It can be characterised by hyperglycaemia (high blood sugar) due to impaired insulin secretion and insulin resistance, with most affected people being overweight or obese [1]. Impaired glucose tolerance (IGT) and impaired fasting glucose, together known as impaired glucose regulation (IGR) or prediabetes, characterise an intermediate condition before converging towards diabetes. Recent studies show that a complex interplay of genetic susceptibility, environmental factors, lifestyle (including diet, physical activity, smoking and alcohol consumption), clinical heterogeneity, drugs and gut microbiome orchestrates the development of type 2 diabetes [2]. Over time, individuals with type 2 diabetes are more likely to have a higher risk for heart attacks, strokes [3], neuropathy (nerve damage), retinopathy (causing blindness) and kidney failure as well as several infectious diseases including COVID-19, reducing life quality and causing social burden [4, 5].

Metabolomics profiles involve a set of low-molecular-weight biochemicals (metabolites) that includes sugars, amino acids, organic acids, nucleotides, lipids, xenobiotics and other compound classes. Identifying biochemical changes occurring between prediabetes and diabetes improves risk prediction for better-targeted prevention [6, 7]. In addition, genetic composition can be used to make predictions regarding disease susceptibility. Genome-wide association studies (GWAS) show that more than 400 loci influence the risk of type 2 diabetes [8] and that 900 genetic variants have been associated with BMI [9]. Therefore, linking metabolites with genetics gives access to genetics’ influence on the metabolic compositions [10–13], providing comprehensive molecular understanding of the disease.

In the Innovative Medicines Initiative - Diabetes Research on Patient Stratification (IMI-DIRECT), we characterised 132 metabolites from targeted measurements and 779 metabolites from untargeted measurements profiled in 3000 individuals at baseline. The study population was stratified by following ADA 2011 glycaemic categories as follows: 23.89% (*n*=692) had normal glucose regulation (NGR) with fasting glucose 5.23 (SD=0.39) mmol/l; 48.91% (*n*=1418) had IGR with fasting glucose 5.90 (SD=0.51) mmol/l; and 27.2% (*n*=890) had type 2 diabetes with fasting glucose 7.15 (SD=1.39) mmol/l [14]. For the integration of non-omics data such as health status, lifestyle and medication with metabolomics, advanced statistical techniques were applied to analyse the data (see Methods). Beyond multivariate and association analyses we performed causal mediation analysis to evaluate potential causal roles of mediators on outcome [15, 16]. A study on drug–omics associations in type 2 diabetes [17] used an unsupervised deep learning framework of multi-omics variational autoencoders (MOVE) to extract significant drug response patterns from 789 individuals newly diagnosed with type 2 diabetes in the IMI-DIRECT cohort. We integrated the polypharmacy effect on metabolomics knowledge from MOVE and compared with our molecular findings in this study.

Our aims in this study were as follows: (1) to characterise 911 small molecular (132 targeted, 779 untargeted metabolomics analysis approach) features associated with prediabetes/IGR and type 2 diabetes; (2) to identify baseline metabolites associated with progression rate estimated from cross-sectional data; (3) to investigate potential mediation effects of metabolites from baseline glycaemic status to follow-up using mediation analysis; and (4) to identify causal relationships between metabolites and type 2 diabetes using genetics drivers using two-sample Mendelian randomisation (2SMR) tests.

## Methods

### DIRECT cohort

The Diabetes Research on Patient Stratification (DIRECT) cohort encompasses 24,682 European participants at varying risk of glycaemic deterioration, identified and enrolled into a prospective cohort (study 1) of prediabetes (*n*=2235) and type 2 diabetes (*n*=830). Using ADA 2011 glycaemic categories in study 1, 33% (*n*=692) of cohort 1 (prediabetes risk) had NGR, 67% (*n*=1418) had IGR and 108 were excluded. In study 2, 789 samples were included and 41 samples were excluded. From study 1, 101 excluded samples entered study 2 (*n*=890). The ratio of self-reported sex varied in each study. Detailed characteristics on inclusion and exclusion criteria, along with the protocol timeline for visits and tests for both studies, have been described elsewhere [14, 18]. In summary, venous blood fasting samples were obtained, followed by performance of DNA extractions and additional biochemical analyses. Metabolomics measurements for distinct samples at the baseline is considered in this study.

### Targeted metabolomics (Absolute*IDQ* p150 Kit)

Blood samples in the study were analysed with the Absolute*IDQ* p150 Kit (BIOCRATES Life Sciences, Innsbruck, Austria) (see electronic supplementary material [ESM] Methods for details) [19]. After data export, lower and upper outliers were defined as samples with >33% of metabolite concentrations below 25% quantile (±1.5 × IQR). Metabolite traits with too many zero-concentration samples and unidentified metabolites (NAs, >50%) were excluded (none). The CV was calculated in reference samples for each metabolite over all plates. Metabolite traits with CV>0.25 were excluded. After quality control, 132 metabolites were included in this study (ESM Table 1). Metabolite concentrations were log_*e*_-transformed and scaled (mean=0, SD=1) to ensure comparability between the metabolites.

### Untargeted metabolomics (Metabolon platform)

Untargeted LC/MS-based techniques covers a broad spectrum of metabolites, in contrast to the targeted techniques wherein metabolites are limited to a predefined set of molecules. For details on sample preparation, measurement and identification of metabolites, see ESM Methods. Incomplete databases and the presence of unknown or novel metabolites have been reported with an asterisk (*) against the metabolite name. The measured volume of the datasets contained 12% missing values. We screened for outlier remover (see ESM Fig. 1 for an example), which added 4% more missing values onto existing missing values (ESM Table 2). Peaks were quantified using AUC. For studies spanning multiple days, a data normalisation step was performed to correct variation resulting from instrument inter-day tuning differences. Essentially, each compound was corrected in run-day blocks by registering the medians to equal one and normalising each data point proportionately (termed the ‘block correction’; ESM Fig. 2). Principal component analysis was performed on the metabolite dataset and checked for technical effects such as centre and sex (see ESM Fig. 3). The data missing pattern was tested using logistic regression considering missing as 0 and non-missing as 1; there was no significant association between missing and regressors indicating the missing-at-random pattern. The K-nearest neighbour (KNN)-based imputation method was applied using K=10 as suggested and optimised from German Cohort KORA F4 [20].

### Statistics

#### Multivariable logistic regression and linear regression

Identifying metabolites specifically associated with the presence of IGR and type 2 diabetes, we ran the logistic regression with adjustment for age, sex, BMI and centre as the basic model, and adjusted additionally for alcohol consumption, smoking, BP, fasting HDL-cholesterol and fasting triacylglycerol as the full model. The concentration of each metabolite was log_*e*_-transformed and scaled to have a mean of zero and an SD of 1. Each metabolite was taken as exposure and a binary NGR-IGR, NGR-type 2 diabetes (NGR-T2D) or IGR-type 2 diabetes (IGR-T2D) variable as an outcome. The OR of outcomes was calculated using the β coefficient from logistic regression, where OR>1 indicates higher odds of outcome and OR<0 shows lower odds of outcome. To account for multiple testing, the *p* values from regression analyses were adjusted for multiple testing using the Bonferroni correction (*p*_*fdr*_ values). To stratify sex-dependent metabolites, men and women were separated to test the associations by performing the logistic regression full models.

For incidents of IGR and type 2 diabetes analysis, a binary NGR-IGR, NGR-T2D or IGR-T2D variable at follow-up times of 18 months and 48 months was taken as the outcome; transformed metabolites and the same risk factors in the full model were taken as exposure and covariates, respectively. The same *p* correction method was adopted.

The linear regression model was used to explore the association between HbA_1c_ progression rate and metabolites at the baseline. HbA_1c_ progression rate was computed with a conditional linear mixed effect model and adjusted for changes in BMI and diabetes medications [21]. Each transformed metabolite was taken as the independent variable and HbA_1c_ concentration as the dependent variable, with adjustment for age and sex. Bonferroni correction was performed for *p* correction.

#### Mediation analysis

Mediation analysis followed the basic steps suggested by Baron and Kenny [22], and the significance of the mediation effect was tested with a non-parametric causal mediation analysis [22, 23]. Each identified metabolite was taken as a mediator, glycaemic category status at the baseline as the independent variable and glycaemic category at the follow-up (18 months and 48 months) as the dependent variable. R package ‘mediation (4.5.0)’ was used to calculate the *p* value and proportion of the mediation effect by bootstrapping with 1000 resamples.

#### Mendelian randomisation

We used 2SMR approaches from the MRInstruments (0.3.2) and TwoSampleMR library (v0.5.6) to check causal inference [24]. The 2SMR technique enables the establishment of a causal relationship between two observational studies (ESM Fig. 4), solely relying on summary statistics obtained from GWAS [24, 25]. To evaluate the influence of type 2 diabetes on metabolite levels, we conducted a 2SMR examination. Type 2 diabetes instruments were obtained from the genome-wide genotyping study [26] and the corresponding SNP estimates on metabolites were extracted from the metabolite-GWAS [10, 27]. Prior to performing Mendelian randomisation (MR) analysis, exposure and outcome data were harmonised by aligning the SNPs on the same effect allele. We employed the inverse‐variance weighting [10, 26, 27] to estimate the causal effect.

## Results

### Study populations

After stringent quality control (see ESM Methods), we identified 132 (ESM Table 1) and 779 (ESM Table 2) metabolites from targeted and untargeted metabolomics measurements, respectively, that were profiled for 3000 samples (ESM Table 3) [28]. Baseline characteristics (Table 1) revealed that there were significant differences in BMI, fasting variables and health status observed between NGR, IGR and type 2 diabetes groups. No significant differences in age and smoking status were observed between these three groups. In addition, the study was conducted across seven countries; type 2 diabetes participants were recruited in all centres while participants with NGR or IGR were only recruited in the Amsterdam, Copenhagen, Kuopio and Lund centres.

**Table 1:** Baseline characteristics of the DIRECT participants based on their glycaemic category

Characteristic	NGR	IGR	T2D	*p* value
Sample size	692	1418	890	
Male sex	519 (75.0)	1074 (75.7)	525 (59.0)	<0.001
Centre				<0.001
Amsterdam	167 (24.1)	300 (21.2)	183 (20.6)	
Copenhagen	54 (7.8)	223 (15.7)	97 (10.9)	
Dundee	0	0	164 (18.4)	
Exeter	0	0	142 (16.0)	
Kuopio	407 (58.8)	820 (57.8)	34 (3.8)	
Lund	64 (9.2)	75 (5.3)	104 (11.7)	
Newcastle	0	0	166 (18.7)	
Age, years	62.15±6.43	62.08±6.19	61.99±7.96	0.894
BMI, kg/m^2^	27.15±3.65	28.33±4.06	30.59±4.92	<0.001
Systolic BP, mmHg	128.48±15.21	131.62±15.20	132.02±15.78	<0.001
Diastolic BP, mmHg	79.18±8.73	81.20±8.97	76.48±9.88	<0.001
Fasting glucose, mmol/l	5.23±0.39	5.90±0.51	7.13±1.39	<0.001
Fasting HDL-cholesterol, mmol/l	1.37±0.35	1.30±0.36	1.18±0.38	<0.001
Fasting LDL-cholesterol, mmol/l	3.21±0.90	3.19±0.95	2.43±1.00	<0.001
Fasting TG, mmol/l	1.22±0.53	1.44±0.66	1.56±0.88	<0.001
Fasting cholesterol, mmol/l	5.14±0.97	5.15±1.01	4.33±1.17	<0.001
Fasting HbA_1c_, mmol/mol	35.34±2.22	37.86±2.88	45.86±5.94	<0.001
Fasting HbA_1c_, %	5.38±0.20	5.61±0.26	6.35±0.54	<0.001
Fasting insulin, pmol/l	50.84±30.90	72.42±50.22	96.56±72.69	<0.001
Smoking status				0.717
Current smoker	93 (13.4)	215 (15.2)	117 (13.2)	
Ex-smoker	326 (47.1)	681 (48.0)	445 (50.1)	
Never	272 (39.3)	520 (36.7)	326 (36.7)	
Not Known	1 (0.1)	2 (0.1)	1 (0.1)	
Alcohol consumption status				0.004
Never	96 (13.9)	166 (11.7)	140 (15.7)	
Occasionally	134 (19.4)	282 (19.9)	214 (24.1)	
Regularly	462 (66.8)	968 (68.3)	534 (60.1)	
Not known	0	2 (0.1)	1 (0.1)	
Health status				<0.001
Poor	1 (0.1)	10 (0.7)	28 (3.1)	
Fair	49 (7.1)	74 (5.2)	34 (3.8)	
Good	331 (47.8)	744 (52.5)	428 (48.1)	
Very good	213 (30.8)	396 (27.9)	239 (26.9)	
Excellent	49 (7.1)	74 (5.2)	34 (3.8)	
Not known	4 (0.6)	11 (0.8)	19 (2.1)	

Quantitative variables are expressed as mean ± SD; categorical variables are expressed as *n* (%)

The significant difference of population characteristics between the individuals with IGR/type 2 diabetes and the normal participants (NGR) was calculated. Test statistics for categorical variables were calculated via the *χ*^2^ test and Student’s *t* test for continuous variables

T2D, type 2 diabetes; TG, triacylglycerol

### Metabolites associated with prediabetes and diabetes from targeted metabolomics measurements

**Fig. 1:**
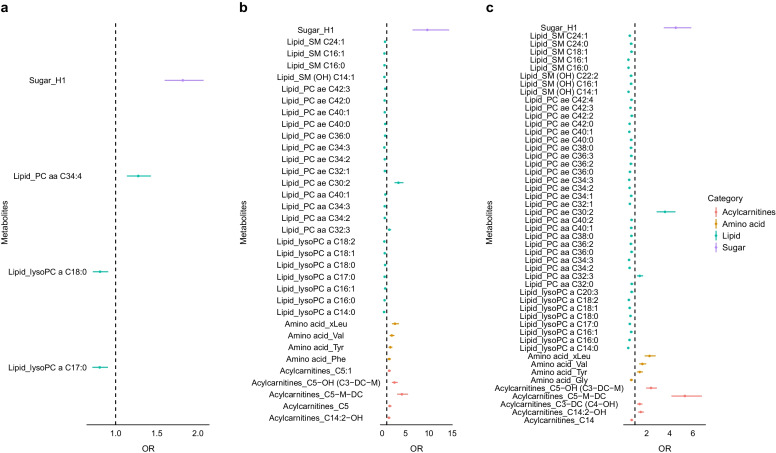
Flag plots representing the results of the multivariable logistic regression models for NGR vs IGR (**a**), NGR vs type 2 diabetes (**b**) and IGR vs type 2 diabetes (**c**) as dependent variables and the metabolites as independent variables, adjusted for study centre, sex, age, BMI, BP, fasting HDL-cholesterol, fasting triacylglycerol, smoking status, alcohol status and health status. The *x*-axis shows OR (95% CI) and the *y*-axis shows each significant metabolite; metabolite classes are represented by different colours. SM, sphingomyelin

A multivariable logistic regression model was used with known diabetes-related variables as covariates to identify significant metabolites. Study centre, sex, age and BMI were covariates in the basic model while the additional variables systolic BP, fasting HDL-cholesterol, fasting triacylglycerol, smoking status, alcohol status and health status were added in the full model. Based on the full model, four metabolites differed significantly between the NGR and IGR groups (Fig. 1a). Of these, hexoses (H1) showed the strongest association (OR 1.81 [95% CI 1.59, 2.06], *p*_*fdr*_=3.97×10^−17^) and served as a positive control throughout our analysis. Thirty-four and 50 metabolites differed significantly between NGR and IGR vs type 2 diabetes, respectively (Fig. 1b,c). As a general pattern, phosphatidylcholines (PCs) and lysophosphatidylcholine (lysoPC) were negatively associated with progression to type 2 diabetes, while branched-chain and aromatic amino acids as well as valeryl/glutaryl-related acylcarnitines were positively associated with type 2 diabetes.

H1 (OR 9.67 [95% CI 6.54, 14.32], *p*_*fdr*_=1.13×10^−27^) also had the strongest associations in NGR-T2D while C5-M-DC (OR=5.31 [95% CI 4.16, 6.77], *p*_*fdr*_=1.07×10^−38^) had the strongest association in IGR-T2D. Three metabolites (H1, lysoPC a C17:0, lysoPC a C18:0) were significantly different in all comparisons (NGR-IGR, NGR-T2D and IGR-T2D), suggesting their important roles in diabetes indication and severity. Detailed statistics for the basic model and full model are shown in ESM Tables 3–8. As there were many more male participants than female participants enrolled in the study, a sensitivity analysis stratified by sex was conducted, and is reported in ESM Results, ESM Tables 9–14 and ESM Fig. 5.

### Metabolites associated with prediabetes and diabetes from untargeted metabolomics measurements

**Fig. 2:**
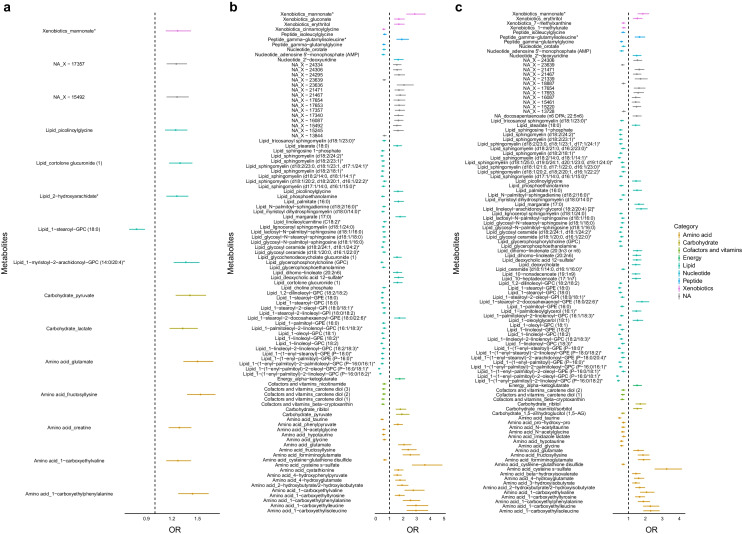
Flag plots representing the results of the multivariable logistic regression models for NGR vs IGR (**a**), NGR vs type 2 diabetes (**b**) and IGR vs type 2 diabetes (**c**) as dependent variables and the metabolites as independent variables, adjusted for study centre, sex, age, BMI, BP, fasting HDL-cholesterol, fasting triacylglycerol, smoking status, alcohol status and health status. The *x*-axis shows OR (95% CI) and the *y*-axis shows each significant metabolite; metabolite classes are represented by different colours. Asterisks (*) indicate the presence of unknown or novel metabolites

Fifteen metabolites were significantly changed between NGR and IGR based on the logistic regression analyses in the full model (Fig. 2a). Fructosyl lysine had the highest statistically significant association with progression to IGR (OR 1.53 [95% CI 1.37, 1.71], *p*_*fdr*_=8.64×10^−12^). Similarly, 99 and 108 metabolites differed significantly between NGR or IGR and type 2 diabetes, respectively (Fig. 2b,c). As a general pattern, lipids were negatively associated and amino acids were positively associated with progression to type 2 diabetes. 1-(1-Enyl-palmitoyl)-2-oleoyl-GPC (P-16:0_18:1)* (OR 0.23 [95% CI 0.17, 0.31], *p*_*fdr*_=3.48×10^−18^) had the strongest association for the NGR-T2D comparison, while cysteine-*S*-sulphate (OR 3.25 [95% CI 2.55, 4.15], *p*_*fdr*_=3.11×10^−18^) was significantly associated in the IGR-T2D comparison. Seven metabolites (fructosyl lysine, glutamate, 1-stearoyl-GPC (18:0), *N*-lactoylphenylalanine, *N*-lactoylvaline, picolinoyl glycine, mannonate) appeared significant in all comparison groups, suggesting their important roles as diabetes risk indicators. Detailed statistics are presented in ESM Tables 15–20. A sex-based sensitivity analysis of metabolomics data from the untargeted measurements is reported in ESM Results, ESM Table 21–26, ESM Fig. 6.

### Metabolites associated with HbA_1c_ progression rate

HbA_1c_ progression rate was computed with a conditional linear mixed effect model and adjusted for changes in BMI and diabetes medications [21]. In multivariable linear regression analysis, lysoPC a C17:0 (β −0.0535 [95% CI −0.08, −0.0269], *p*_*fdr*_=0.0109), glycine (Gly) (β −0.0509 [95% CI −0.0782, −0.0236], *p*_*fdr*_=0.0347) and H1 (β 0.0481 [95% CI 0.0218, 0.0745], *p*_*fdr*_=0.0452) were significantly correlated with HbA_1c_ progression rate and all were related to glycaemic-deterioration traits as well. In untargeted metabolomic profiling, 20 metabolites were significantly related to HbA_1c_ progression rate, with pyruvate (β 0.0877 [95% CI 0.0609, 0.114], *p*_*fdr*_=1.28×10^−7^) showing the strongest association. Besides pyruvate, *N*-lactoylleucine, lactate, *N*-lactoylphenylalanine, X-15245, *N*-lactoylisoleucine, *N*-lactoylvaline, 1-(1-enyl-palmitoyl)-2-oleoyl-GPC (P-16:0/18:1)*, cortolone glucuronide, X-24295, formiminoglutamate and *N*-lactoyltyrosine were also significantly associated with glycaemic categories. Tables 2 and 3 show the metabolites with significant associations, while the complete results are reported in ESM Tables 27–28.

**Table 2:** Metabolites from targeted measurements significantly associated with HbA_1c_ progression rate from a linear regression model

Metabolite	β (95% CI)	*p* value	*p*_*fdr*_ value
LysoPC a C17:0	−0.053 (−0.080, −0.027)	8.25×10^−5^	0.011
Gly	−0.051 (−0.078, −0.024)	2.63×10^−4^	0.0345
H1	0.048 (0.022, 0.075)	3.42×10^−4^	0.045

The dependent variable is the HbA_1c_ progression rate while the independent variable is the log_*e*_-transformed and standardised baseline concentration of a given metabolite, adjusted by age and sex

The *p*_*fdr*_ values represent the adjusted* p* value for multiple testing by Bonferroni correction

**Table 3:** Metabolites from untargeted metabolomics measurements significantly associated with HbA_1c_ progression rate from a linear regression model

Metabolite	β (95% CI)	*p* value	*p*_*fdr*_ value
Pyruvate	0.087 (0.060, 0.114)	1.65×10^−10^	1.28×10^−7^
*N*-Lactoylleucine	0.082 (0.056, 0.109)	8.43×10^−10^	6.57×10^−7^
Lactate	0.075 (0.049, 0.102)	3.30×10^−8^	2.57×10^−5^
*N*-Lactoylphenylalanine	0.074 (0.048, 0.100)	3.66×10^−8^	2.85×10^−5^
X-15245	0.074 (0.047, 0.100)	6.24×10^−8^	4.86×10^−5^
*N*-Lactoylisoleucine	0.068 (0.042, 0.095)	3.11×10^−7^	2.42×10^−4^
*N*-Lactoylvaline	0.067 (0.041, 0.094)	5.69×10^−7^	4.43×10^−4^
X-11444	0.068 (0.041, 0.094)	6.22×10^−7^	4.84×10^−4^
Orotidine	0.065 (0.038, 0.091)	1.74×10^−6^	1.35×10^−3^
Metabolonic lactone sulphate	0.063 (0.036, 0.089)	2.9 ×10^−6^	2.28×10^−3^
3,4-Dihydroxybutyrate	0.060 (0.033, 0.087)	1.11×10^−5^	8.64×10^−3^
*N*4-Acetylcytidine	0.059 (0.033, 0.085)	1.16×10^−5^	9.06×10^−3^
X-24337	0.058 (0.032, 0.085)	1.47×10^−5^	0.011
1-(1-Enyl-palmitoyl)-2-oleoyl-GPC(P-16:0/18:1)*	−0.058 (−0.084, −0.032)	1.49×10^−5^	0.016
X-25828	−0.058 (−0.085, −0.032)	1.50×10^−5^	0.017
Cortolone glucuronide	0.058 (0.032, 0.085)	1.73×10^−5^	0.013
X-24295	0.057 (0.031, 0.084)	1.77×10^−5^	0.014
Formiminoglutamate	0.059 (0.032, 0.088)	2.75×10^−5^	0.021
1-Palmitoyl-2-oleoyl-GPE (16:0/18:1)	0.056 (0.029, 0.082)	3.59×10^−5^	0.028
*N*-Lactoyltyrosine	0.055 (0.029, 0.082)	3.98×10^−5^	0.031

The dependent variable is the HbA_1c_ progression rate while the independent variable is the log_*e*_-transformed and standardised baseline concentration of a given metabolite, adjusted by age and sex. The *p*_*fdr*_ are adjusted *p* for multiple testing by Bonferroni correction

### Metabolite association with incident diabetes (IGR/type 2 diabetes)

Several metabolites were identified to be significantly associated with HbA_1c_ progression rate as well as glycaemic category: three targeted metabolites (lysoPC a C17:0; glycine, H1); and 12 untargeted metabolites (pyruvate, *N*-lactoylleucine, lactate, *N*-lactoylphenylalanine, X-15245, *N*-lactoylisoleucine, *N*-lactoylvaline, 1-[1-enyl-palmitoyl[-2-oleoyl-GPC* [PC(P-16:0/18:1)], cortolone glucuronide, X-24295, formiminoglutamate, *N*-lactoyltyrosine). Next, we investigated their predictive value for IGR and type 2 diabetes by including baseline metabolite concentrations and incident IGT or type 2 diabetes in follow-up timelines in multivariable logistic regression. As shown in Table 4, lysoPC a C17:0 concentration at baseline was observed to significantly differ in 244 incident IGR individuals compared with 398 NGR control individuals after 18 months. The sum of H1 at baseline concentrations showed significant differences between incident IGR (at 48 month follow-up) and NGR or incident type 2 diabetes and IGR at both the 18 month and the 48 month follow-up.

**Table 4:** Metabolites from targeted measurements that were significantly associated with incidence of IGR and type 2 diabetes in different pairwise comparisons

Comparison	OR (95% CI)	*p* value
18 months		
398 NGR vs 244 IGR		
lysoPC a C17:0	−0.246 (−0.452, −0.043)	0.018
897 IGR vs 71 T2D		
H1	0.545 (0.164, 0.945)	0.006
48 months		
244 NGR vs 295 IGR		
H1	0.433 (0.189, 0.690)	7x10^−3^
821 IGR vs 128 T2D		
H1	0.347 (0.064, 0.642)	0.018

Baseline metabolites were taken as the independent variables with glycaemic category in different timelines (18 months and 48 months) as the dependent variables, adjusted by study centre, sex, age, BMI, BP, fasting HDL-cholesterol, fasting triacylglycerol, smoking status, alcohol status and health status

ORs and *p* values were calculated from the logistic regression model

T2D, type 2 diabetes

In untargeted metabolomic profiling, lactate and X-24295 baseline concentrations were significantly correlated with IGR or type 2 diabetes incidence at the 18 month and 48 month follow-up (Table 5). Formiminoglutamate, *N-*lactoylleucine and *N-*lactoylvaline significantly differed in 244 incident IGT individuals compared with 398 NGT control individuals after 18 months. We did not find any significant metabolites from untargeted measurements to predict the incidence of IGR from NGR at 48 months.

**Table 5:** Metabolites from untargeted measurements that were significantly associated with incidence of IGR and type 2 diabetes in different pairwise comparisons

Comparison	OR (95% CI)	*p* value
18 months		
398 NGR vs 244 IGR		
Formiminoglutamate	0.369 (0.157, 0.588)	7.7×10^−4^
Lactate	0.373 (0.143, 0.557)	0.002
*N*-Lactoylleucine	0.294 (0.079, 0.514)	0.008
*N*-Lactoylvaline	0.248 (0.039, 0.460)	0.021
X-24295	0.225 (0.022, 0.432)	0.031
897 IGR vs 71 T2D		
X-24295	0.474 (0.162, 0.801)	3.6x10^−3^
Lactate	0.409 (0.077, 0.747)	1.6x10^−2^
48 months		
821 IGR vs 128 T2D		
X-24295	0.474 (0.162, 0.801)	3.6x10^−3^
Lactate	0.409 (0.077, 0.747)	1.6x10^−2^

Baseline metabolites were taken as the independent variables with glycaemic category in different timelines (18 months and 48 months) as the dependent variables, adjusted by study centre, sex, age, BMI, BP, fasting HDL-cholesterol, fasting triacylglycerol, smoking status, alcohol status and health status

ORs and *p* values were calculated from the logistic regression model

T2D, type 2 diabetes

### Mediation analysis

**Fig. 3:**
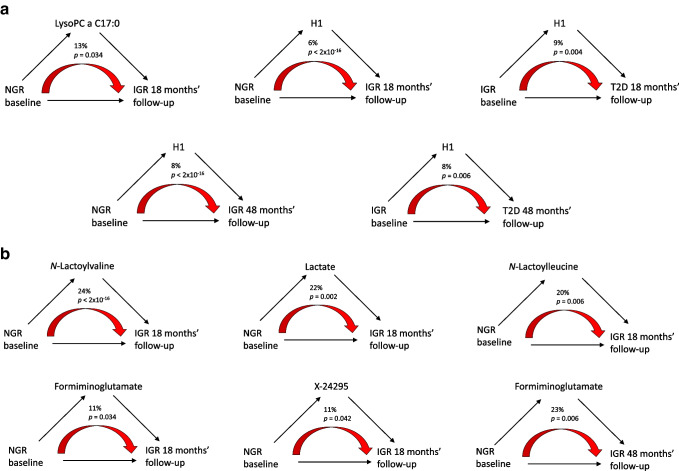
Schematic overview of mediation analysis with lysoPC a C17:0 and hexoses (**a**) or *N*-lactoylvaline, lactate, *N*-lactoylleucine, formiminoglutamate and X-24295 (**b**) as mediators. Numbers above the red arrows indicate the percentage and significance of mediation effects. T2D, type 2 diabetes

Causal mediation analysis was employed to explore the potential mediation effects of the identified metabolites from baseline glycaemic status to follow-up. Consistent with incidence results, lysoPC a C17:0 showed strong significance (proportion of mediation by 13%, mediation effect *p*=0.034, Fig. 3a), indicating that this metabolite partially mediated the glycaemic deterioration from NGR to IGR at 18 months. The positive control H1 exhibited significant mediation effects in all groups (between 6% and 9%) as it is mainly represented by blood glucose.

*N*-Lactoylvaline (proportion of mediation 24%, mediation effect *p*<2×10^−16^), lactate (proportion of mediation 22%, mediation effect *p*=0.002), *N*-lactoylleucine (proportion of mediation 20%, mediation effect *p*=0.006), formiminoglutamate (proportion of mediation 11%, mediation effect *p*=0.034) and X-24295 (proportion of mediation 11%, mediation effect *p*=0.042) were all observed to show significant mediation effects from baseline NGR to IGR at 18 months’ follow-up (Fig. 3b). Furthermore, formiminoglutamate (proportion of mediation 23%, mediation effect *p*=0.006) showed a significant mediation effect from NGR to IGR at 48 months. These results suggest that these metabolites own a significant mediation effect on glycaemic deterioration.

### MR

**Fig. 4:**
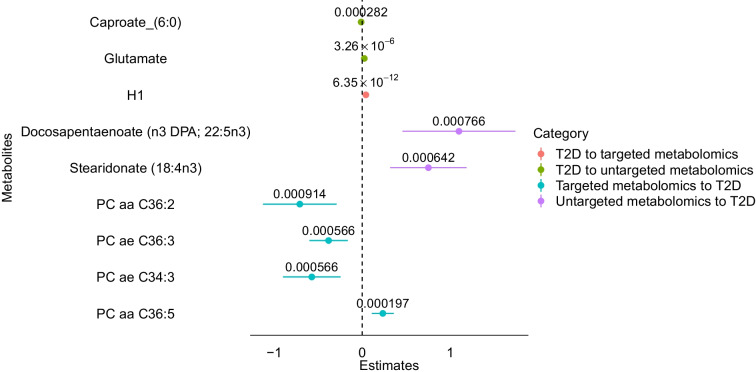
Forest plot representing causal estimates of type 2 diabetes on targeted and untargeted metabolites in the two-sample MR test. T2D, type 2 diabetes

The availability of genetic data on type 2 diabetes makes the use of MR particularly compelling. To assess bidirectional causal relationships between type 2 diabetes and metabolites (Fig. 4), we employed 2SMR tests. After multiple testing correction only the concentration of the sum of H1 was determined by type 2 diabetes (*p*<0.05/117=0.00042). For untargeted metabolites we found instruments for only 19% of the metabolites (i.e. 151 out of 779). For example, instruments are from genes *TCF7L2*, *IGF2BP2*, *NOTCH2*, *CDKAL1*, *PABPC4*, *FTO* and *JAZF1*, known to be associated with diabetes and that have been further significantly associated with the metabolites. Following multiple testing correction, it suggests that the change in an amino acid (glutamate) and a lipid (caproate, FA C6:0) was caused by change in type 2 diabetes status (*p*<0.05/151=0.000331). However, metabolites that are causal for type 2 diabetes (meaning that the change in metabolite caused change in the disease status) included several phosphatidylcholines, namely PC aa C36:2, PC aa C36:5, PC ae C36:3 and PC ae C34:3, from the targeted metabolomics dataset. From the untargeted metabolomics dataset, two *n*-3 fatty acids, namely stearidonate (18:4n3) and docosapentaenoate (n3 DPA; 22:5n3), were identified to be causal for type 2 diabetes. Detailed statistics of our MR analysis are presented in ESM Tables 29–32.

## Discussion

In this study, we used untargeted metabolomics to provide semi-quantitative global screening of metabolites in the development of a disease whereas targeted metabolomics was used to quantify a pre-selected subset of metabolites with absolute concentrations. However, the overlap between the two metabolomic techniques was limited to a few amino acids and lipids. In the current study we report 19 metabolites (three from targeted and 14 from global profiling, plus one common lysoPC a C18:0 / 1-stearoyl-GPC [18:0]) that were significantly associated with prediabetes in the DIRECT cohort. The advantages of global profiling become evident as it allows for the identification of a broader spectrum of metabolites. Few notable examples are given here. First, picolinoylglycine (HMDB0059766), which is potentially a phase II product of picolinic acid, a degradation product of tryptophan [29] and glycine [30], and shows potential as a novel marker for glycaemic deterioration. Prediabetes is often associated with dyslipidaemia, marked by an imbalanced lipid profile compared with individuals with NGR [24]. Second, *N-*lactoyl amino acids are not infrequently observed in metabolomic datasets. In fact it has come to light that *N*-lactoyl amino acids were misidentified in some metabolomic studies and were erroneously reported as 1-carboxyethyl amino acids. In particular, *N*-lactoyl-phenylalanine (Lac-Phe) is known to act as an appetite suppressant when given to obese mice [31]. However, in humans Lac-Phe concentrations were observed to rise following vigorous exercise [32]. In fact, the most recent study shows that Lac-Phe facilitates the impact of metformin on both food intake and body weight [33, 34]. It seems that the exact role of Lac-Phe in the human body and pathways downstream, such as energy metabolism, insulin signalling, exercise-induced pathways, are unclear and needs further research.

We are aware of several limitations to our study. Although metabolomics screening showcases numerous valuable attributes in health science, challenges inherent to this approach continue to exist, especially in the accurate identification of metabolites which is crucial for the biological interpretation and validation of metabolomics data [35]. Variability in sample collection, preparation and analytical techniques can impact the reproducibility and comparability of results across different studies. Standardisation efforts are ongoing but may not fully address all sources of variation. The identification of metabolites, especially in untargeted metabolomics, can be challenging. Incomplete databases and the presence of unknown or novel metabolites have been reported with a metabolite name with an asterisk (*) sign. However, ongoing advancements in technology, methodology and standardisation efforts aim to enhance the robustness and applicability of metabolomics studies [35]. The current study is predominantly based on White male participants from the Kuopio region of Europe, and for this reason an additional sex-based sensitivity analysis has been performed and reported separately (ESM Results 1 and 2). Challenges in MR studies include limited statistical power, potential reverse causation, confounding and pleiotropy [36]. Caution is advised in interpreting causality inference, considering the various limitations mentioned in the methods, and precautionary measures were taken by using valid MR instruments and reporting Bonferroni significance.

A drug–metabolomics associations study [17] was examined to determine whether or not metabolites linked to type 2 diabetes from the DIRECT study were also associated with a particular drug. Looking at our results and those of Allesøe et al [17], we found that 44% (15 out of 34) of targeted metabolites and 3% (three out of 99) of non-targeted metabolites that were significantly associated with type 2 diabetes also showed a significant association with at least one of the 20 drugs. This suggests that metabolites linked to type 2 diabetes may be confounded by polypharmacy.

However, metabolite association with incident prediabetes or diabetes (IGR-T2D) showed that lysoPC a C17:0 could predict the risk of developing IGR at 18 months and 48 months. It has already been shown that lysoPCs differ significantly between individuals with incident IGT or type 2 diabetes and individuals with NGR in the KORA study [37]. LysoPC a C17:0 was negatively associated with diabetes, a finding that was confirmed in several studies [38, 39]. The aforementioned drug–metabolomics association study [17] showed that lysoPC a 17:0 was not associated with the drugs. However, the origin of odd-chain fatty acids (mainly C15:0 and C17:0) remains elusive. Jenkins et al [40] investigated the origin of circulating odd-chain fatty acids (C17:0, C15:0) through a combination of animal and human studies to determine possible contributions of fatty acids from the gut-microbiota, diet and novel endogenous biosynthesis [41]. The findings suggested that C15:0 was linked to dietary intake, while C17:0 was predominantly biosynthesised, indicating independent origins and non-homologous roles in disease causation.

Causal mediation analysis indicated that plasma lactate strongly mediates the effects of identified metabolites in the transition from baseline glycaemic status to follow-up [42]. In a longitudinal study of Swedish men, elevated serum lactate was independently linked to a higher incidence of type 2 diabetes, irrespective of obesity measures [43]. Formiminoglutamate was confirmed to be associated with a higher risk of incident type 2 diabetes in older Puerto Ricans [44]. *N*-lactoylleucine and *N*-lactoylvaline, derivatives of leucine and valine, respectively, are ubiquitous pseudodipeptides of lactic acid and amino acids that are formed by reverse proteolysis [32] and are correlated with underivatised amino acids in human plasma. The Microbiome and Insulin Longitudinal Evaluation Study (MILES) [45] investigated the association between *ABO* haplotypes and insulin-related characteristics, and explored possible pathways that could mediate these associations. The study showed that the A1 haplotype potentially enhances favourable insulin sensitivity in non-Hispanic White individuals, with lactate likely influencing this mechanism, while gut bacteria are not believed to be a contributing factor.

In MR, causality signifies that modifying exposure leads to a predictable change in the outcome. Our 2SMR analysis suggests that the metabolites causal for type 2 diabetes are PC aa C36:2, PC aa C36:5, PC ae C34:3 and PC ae C36:3 and all these metabolites are significantly associated with drug–metabolomics. However, from untargeted metabolomics two *n*-3 fatty acids, namely stearidonate (18:4n3) and docosapentaenoate DPA 22:5n3), are not further associated with drugs. In 2012, Banz et al [46] explored the therapeutic implications of stearidonate acid in preventing or managing type 2 diabetes. The Fatty Acids and Outcomes Research Consortium (FORCE) [47] found that higher circulating biomarkers of seafood-derived *n*-3 fatty acids were associated with lower type 2 diabetes risk. On the contrary, branched-chain amino acids [48] and sphingomyelin [15] have been shown to have a causal role in type 2 diabetes development, a correlation not observed in the DIRECT study.

## Conclusions

Our study demonstrates that alteration in blood plasma metabolites is associated with glycaemic deterioration. The progression from prediabetes to diabetes is mediated by novel metabolites such as picolinoylglycine and *N-*lactoyl-amino acids, as demonstrated by evidence from the DIRECT study. *N*-lactoyl-amino acids are known to be exercise-induced metabolites that suppress food intake and influence glucose homeostasis. Additional functional research and quantification are needed to advance the identification of early metabolic biomarkers such as *N****-***lactoyl-amino acids, which have the potential to forecast the onset of type 2 diabetes. Collectively, these findings direct attention towards novel metabolic signatures associated with glycaemic deterioration.

## Supplementary Information

Below is the link to the electronic supplementary material.

ESM (PDF 2727 KB)

ESM Tables (XLSX 1294 KB)

Supplementary file3 (R 4 KB)

## Data Availability

Access to the molecular and clinical raw data, as well as the processed data, is restricted. This is in accordance with the informed consent provided by study participants, the various national ethical approvals obtained for the study, and compliance with the European General Data Protection Regulation (GDPR). Individual-level clinical and molecular data cannot be transferred from the centralised IMI-DIRECT repository. Requests for access will receive guidance on accessing data through the DIRECT secure analysis platform after submitting an appropriate application. The IMI-DIRECT data access policy and additional information about the IMI-DIRECT research consortium’s initiatives and activities can be found at https://directdiabetes.org. Code used for MR in the study is included as ESM.

## Abbreviations

2SMR: Two-sample MR
GWAS: Genome-wide association study
H1: Hexoses
IGR: Impaired glucose regulation
IGT: Impaired glucose tolerance
IMI-DIRECT: Innovative Medicines Initiative - Diabetes Research on Patient Stratification
Lac-Phe: *N*-lactoyl-phenylalanine
LysoPC: Lysophosphatidylcholine
MOVE: Multi-omics variational autoencoders
MR: Mendelian randomisation
NA: Unidentified metabolite
NGR: Normal glucose regulation
PC: Phosphatidylcholine
