# Supplementary material for: Role of human plasma metabolites in prediabetes and type 2 diabetes from the IMI-DIRECT study
Source: Diabetologia. 2024 Sep 30;67(12):2804–18. doi: 10.1007/s00125-024-06282-6 (PMC11604760; doi:10.1007/s00125-024-06282-6)
Supplement: Supplementary file 1 — ESM (PDF 2727 KB) [file 125_2024_6282_MOESM1_ESM.pdf]

ESM Methods

1 Targeted Metabolomics

We used a FIA-ESI-MS/MS-based multiple reaction monitoring (MRM) approach to quantify 163 metabolites in 10 µL of EDTA-plasma samples with the Biocrates AbsoluteIDQ p150 Kit (BIOCRATES Life Sciences AG, Innsbruck, Austria). The Kit includes free carnitine (C0), 40 acylcarnitines (Cx:y), 14 amino acids, hexoses (sum of hexoses – about 90-95 % glucose), 92 glycerophospholipids (15 lysophosphatidylcholines (lysoPC) and 77 phosphatidylcholines (PC)), and 15 sphingomyelins (SMx:y). The abbreviations Cx:y is used to describe the total number of carbons and double bonds of all chains, respectively. PCs with two acyl chains are denoted as PC aa (Cx:y), PCs containing an ether bond are denoted as PC ae (Cx:y). Sample preparation and mass spectrometric measurements were performed as described in Römisch-Margl W. et al 2012 (see below) and the manufacturer manual UM-P150. Briefly, sample handling was performed with a Hamilton Microlab STAR robot (Hamilton Bonaduz AG, Bonaduz, Switzerland) and a Ultravap nitrogen evaporator (Porvair Sciences, Leatherhead, U.K.), beside standard laboratory equipment. Mass spectrometric analyses were done on an API 4000 triple quadrupole system (Sciex Deutschland GmbH, Darmstadt, Germany) equipped with a 1200 Series HPLC (Agilent Technologies Deutschland GmbH, Böblingen, Germany) and an HTC PAL auto sampler (CTC Analytics, Zwingen, Switzerland) controlled by the software Analyst 1.6.2. Data evaluation for quantification of metabolite concentrations and quality assessment was performed with the software MultiQuant 3.0.1 (Sciex) and the Biocrates MetIDQ software package. Metabolite concentrations were calculated using internal standards and are reported in µM. In addition to the investigated study samples, five aliquots of a commercial pooled reference plasma (Sera Laboratories International Ltd., Hull, United Kingdom) were analyzed on each kit plate. The results of these reference plasma aliquots were used for calculation of the coefficient of variation (CV) and data normalization. LOD values for metabolites were empirically determined by Biocrates using a large database of independent kit analyses of human plasma samples. More details about analytical kit specifications can be found in sheet AS-p150-7 (request at Biocrates). Metabolites with LOD > 95% of samples: C5-M-DC, C5:1, lysoPC a C14:0, PC ae C30:2, PC ae C42:0

2 Metabolon

2.1 **Sample Preparation:** Samples were prepared using the automated MicroLab STAR® system from Hamilton Company. All samples were maintained at -80°C until processed. Several recovery standards were added prior to the first step in the extraction process for QC purposes. To remove protein, dissociate small molecules bound to protein or trapped in the precipitated protein matrix, and to recover chemically diverse metabolites, proteins were precipitated with methanol under vigorous shaking for 2 min followed by centrifugation. The resulting extract was divided into five fractions: two for analysis by two separate reverse phases (RP)/UPLC-MS/MS methods with positive ion mode electrospray ionization (ESI), one for analysis by RP/UPLC-MS/MS with negative ion mode ESI, one for analysis by HILIC/UPLC-MS/MS with negative ion mode ESI, and one sample was reserved for backup. Samples were placed briefly on a TurboVap® (Zymark) to remove the organic solvent. The sample extracts were stored overnight under nitrogen before preparation for analysis.

2.2 **Ultrahigh Performance Liquid Chromatography-Tandem Mass Spectroscopy (UPLC-MS/MS):** All methods utilized a Waters ACQUITY ultra-performance liquid chromatography (UPLC) and a Thermo Scientific Q-Exactive high resolution/accurate mass spectrometer interfaced with a heated

electrospray ionization (HESI-II) source and Orbitrap mass analyzer operated at 35,000 mass resolution. The sample extract was dried then reconstituted in solvents compatible to each of the four methods. Each reconstitution solvent contained a series of standards at fixed concentrations to ensure injection and chromatographic consistency. One aliquot was analyzed using acidic positive ion conditions, chromatographically optimized for more hydrophilic compounds. In this method, the extract was gradient eluted from a C18 column (Waters UPLC BEH C18-2.1x100 mm, 1.7 µm) using water and methanol, containing 0.05% perfluoropentanoic acid (PFPA) and 0.1% formic acid (FA). Another aliquot was also analyzed using acidic positive ion conditions; however, it was chromatographically optimized for more hydrophobic compounds. In this method, the extract was gradient eluted from the same afore mentioned C18 column using methanol, acetonitrile, water, 0.05% PFPA and 0.01% FA and was operated at an overall higher organic content. Another aliquot was analyzed using basic negative ion optimized conditions using a separate dedicated C18 column. The basic extracts were gradient eluted from the column using methanol and water, however with 6.5mM Ammonium Bicarbonate at pH 8. The fourth aliquot was analyzed via negative ionization following elution from a HILIC column (Waters UPLC BEH Amide 2.1x150 mm, 1.7 µm) using a gradient consisting of water and acetonitrile with 10mM Ammonium Formate, pH 10.8. The MS analysis alternated between MS and data-dependent MS^n scans using dynamic exclusion. The scan range varied slightly between methods but covered 70-1000 m/z. Raw data files are archived and extracted as described below.

2.3 **Data Extraction and Compound Identification: Raw** data was extracted, peak-identified and QC processed using Metabolon's hardware and software. These systems are built on a web-service platform utilizing Microsoft's .NET technologies, which run on high-performance application servers and fiber-channel storage arrays in clusters to provide active failover and load-balancing. Compounds were identified by comparison to library entries of purified standards or recurrent unknown entities. Metabolon maintains a library based on authenticated standards that contains the retention time/index (RI), mass to charge ratio (*m/z)*, and chromatographic data (including MS/MS spectral data) on all molecules present in the library. Furthermore, biochemical identifications are based on three criteria: retention index within a narrow RI window of the proposed identification, accurate mass match to the library +/- 10 ppm, and the MS/MS forward and reverse scores between the experimental data and authentic standards. The MS/MS scores are based on a comparison of the ions present in the experimental spectrum to the ions present in the library spectrum. While there may be similarities between these molecules based on one of these factors, the use of all three data points can be utilized to distinguish and differentiate biochemicals. More than 3300 commercially available purified standard compounds have been acquired and registered into LIMS for analysis on all platforms for determination of their analytical characteristics. Additional mass spectral entries were created for structurally unnamed biochemicals, identified by virtue of their recurrent nature (both chromatographic and mass spectral). These compounds can be identified by future acquisition of a matching purified standard or classical structural analysis.

2.4 **QA/QC:** Several types of controls were analyzed in concert with the experimental samples: a pooled matrix sample generated by taking a small volume of each experimental sample (or alternatively, use of a pool of well-characterized human plasma) served as a technical replicate throughout the data set; extracted water samples served as process blanks; and a cocktail of QC standards that were carefully chosen not to interfere with the measurement of endogenous compounds were spiked into every analyzed sample, allowed instrument performance monitoring and aided chromatographic alignment.

ESM Tables 33 and 34 describe these QC samples and standards. Instrument variability was determined by calculating the median relative standard deviation (RSD) for the standards added to each sample before injection into the mass spectrometers. Overall process variability was determined by calculating the median RSD for all endogenous metabolites (i.e., non-instrument standards) present in 100% of the pooled matrix samples. Experimental samples were randomized across the platform run with QC samples spaced evenly among the injections.

2.5 **Data Extraction and Compound Identification:** Raw data was extracted, peak-identified and QC processed using Metabolon's hardware and software. These systems are built on a web-service platform utilizing Microsoft's .NET technologies, which run on high-performance application servers and fiber-channel storage arrays in clusters to provide active failover and load-balancing. Compounds were identified by comparison to library entries of purified standards or recurrent unknown entities. Metabolon maintains a library based on authenticated standards that contains the retention time/index (RI), mass to charge ratio (m/z), and chromatographic data (including MS/MS spectral data) on all molecules present in the library. Furthermore, biochemical identifications are based on three criteria: retention index within a narrow RI window of the proposed identification, accurate mass match to the library +/- 10 ppm, and the MS/MS forward and reverse scores between the experimental data and authentic standards. The MS/MS scores are based on a comparison of the ions present in the experimental spectrum to the ions present in the library spectrum. While there may be similarities between these molecules based on one of these factors, the use of all three data points can be utilized to distinguish and differentiate biochemicals. More than 3300 commercially available purified standard compounds have been acquired and registered into LIMS for analysis on all platforms for determination of their analytical characteristics. Additional mass spectral entries were created for structurally unnamed biochemicals, identified by virtue of their recurrent nature (both chromatographic and mass spectral). These compounds can be identified by future acquisition of a matching purified standard or classical structural analysis.

2.6 **Curation:** A variety of curation procedures were done to ensure a high-quality data set was available for statistical analysis and data interpretation. The QC and curation processes were designed to ensure accurate and consistent identification of true chemical entities, and to remove those representing system artifacts, mis-assignments, and background noise. Metabolon data analysts use proprietary visualization and interpretation software to confirm the consistency of peak identification among the various samples. Library matches for each compound were checked for each sample and corrected if necessary.

ESM Results

ESM Results 1: Metabolites associated with prediabetes and diabetes from targeted metabolomics measurements (ESM Figure 5a). In females (ESM Figure 5b), no metabolite was significantly changed between NGT and IGR. In males, four metabolites were significantly different between NGR and IGR (ESM Figure 5c): H1 (OR 1.70 [1.47-1.96], p_{fdr} = 9.92x10^{-11}), lysoPC a C18:0 (OR 0.76 [0.67-0.87], p_{fdr} = 8.52x10^{-03}), lysoPC a C16:0 (OR 0.78 [0.68-0.89], p_{fdr} = 0.0359), and lysoPC a C17:0 (OR 0.78 [0.68-0.89], p_{fdr} = 0.0473). Metabolites differed significantly between NGR or IGR and T2D, 19 and 25 respectively. In agreement with results from both sexes, H1 (OR= 9.48 [5.75-15.63], p_{fdr} = 2.49x10^{-16}) was the most strongly associated feature in the NGR-T2D group and C5-MDC (Beta = 1.48 [1.19-1.79], p_{fdr} = 9.20x10^{-20}) had the strongest association in the IGR-T2D group. Sum of hexoses, lysoPC a C17:0, and lysoPC a C18:0 was significantly changed in all groups. The full statistics are shown in ESM Table 9-14.

ESM Results 2: In line with the targeted metabolomics, we performed a sex-based sensitivity analysis on metabolomics data from the untargeted measurements (ESM Figure 6a). In females (ESM Figure 6b), no metabolite was significantly changed between NGT and IGR while nine metabolites were significantly different in the male group. 36 and 51 metabolites differed significantly in females between NGR or IGR and T2D, respectively. Pyruvate (OR = 0.12 [0.06-0.244], p_{fdr} = 3.59x10^{-7}) was the most strongly associated metabolite in NGR-T2D groups and 1-(1-enyl-palmitoyl)-2-oleoyl-GPC (P-16:0/18:1)* (OR = 0.27 [0.18-0.40], p_{fdr} = 3.02x10^{-7}) in IGR-T2D. In males (ESM Figure 6c), 27 and 48 metabolites differed significantly between NGR or IGR and T2D, respectively. 1-(1-enyl-palmitoyl)-2-oleoyl-GPC (P-16:0/18:1)* (OR 0.30 [0.20--0.43], p_{fdr} = 1.06x10^{-7}) was the most strongly associated metabolite in the NGR-T2D group. This is concordant with results from the analysis using the entire set of participants. Cysteine-S-sulfate (OR = 1.03 [0.728-1.36], p_{fdr} = 8.04x10^{-8}) had the strongest association in the IGR-T2D group. Fructosyl-lysine, N-lactoylphenylalanine and 1-stearoyl-GPC (18:0) were significant in all analyses. The full statistics are reported in ESM Table 21-26.

ESM Figures

ESM Figure 1: Outlier example for metabolite X98 on left with outlier on right after removing outliers.

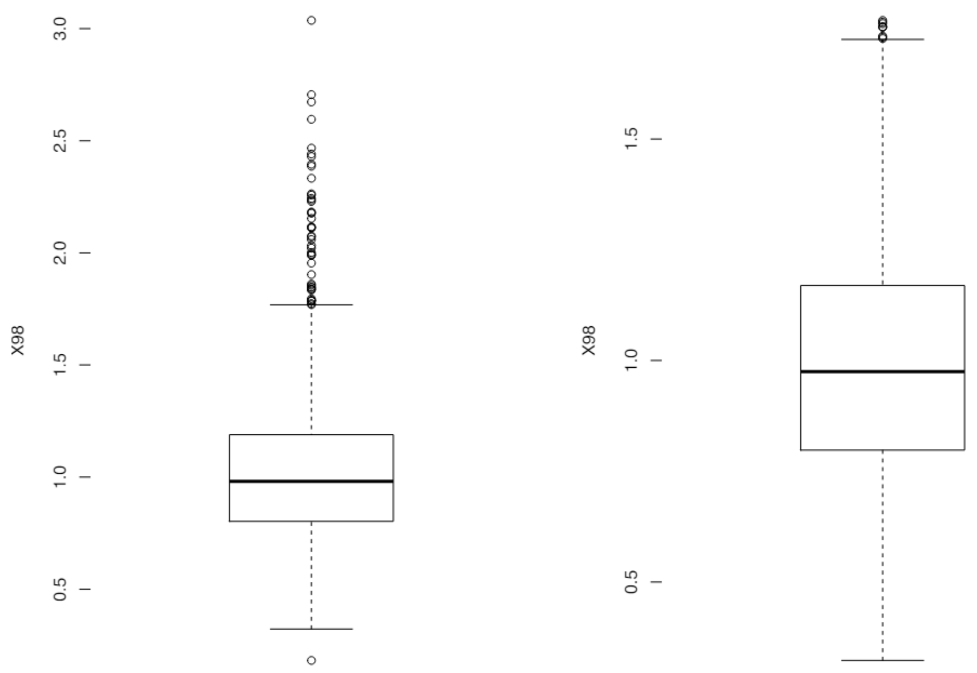

ESM Figure 2: Block correction provided from Metabolon.

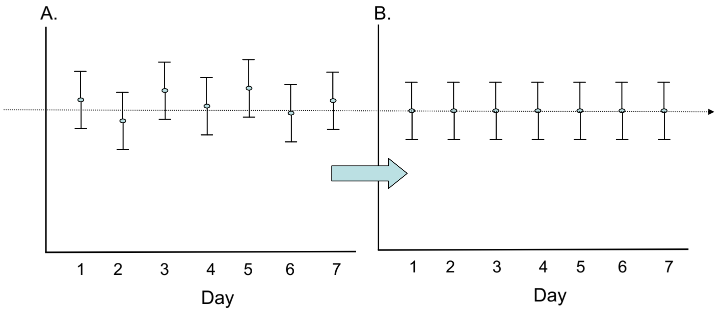

ESM Figure 3: First two principal components plotted as a scatter plot where each point is the sample and variance explained from the metabolites. Color indicated different centers. Both figures show no center effect on the measurement of the untargeted metabolomics.

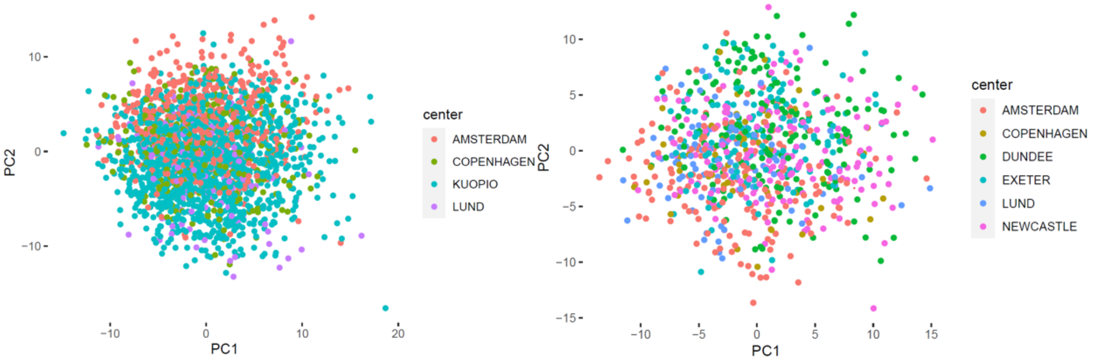

ESM Figure 4. Schematic overview of mendelian randomization

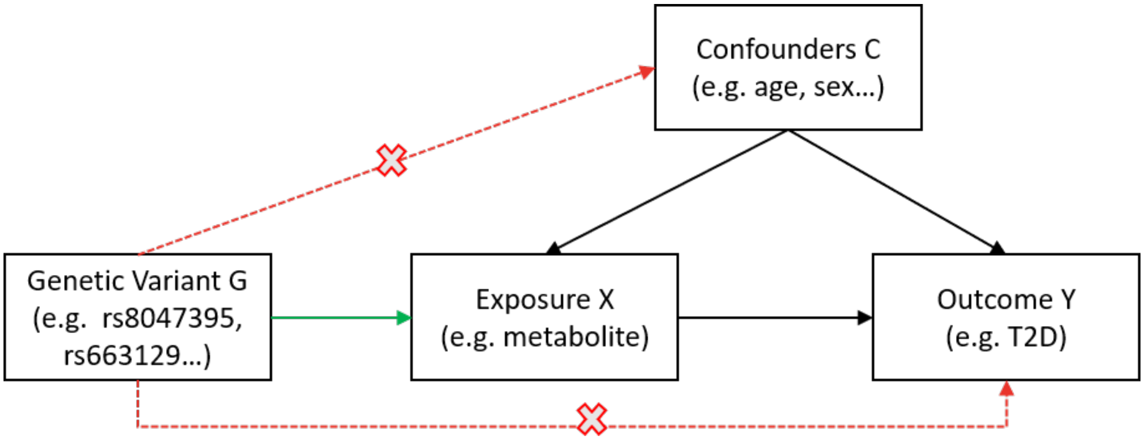

ESM Figure 5: Metabolites associated with prediabetes and diabetes from targeted metabolomics measurement.

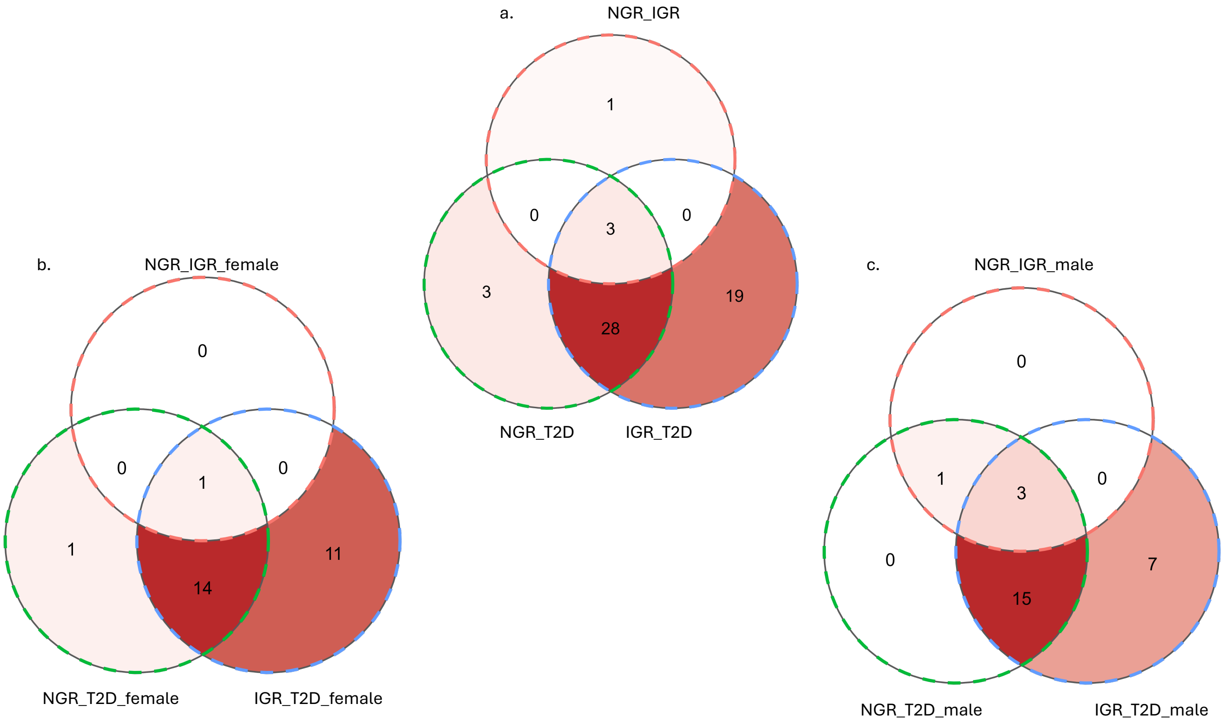

ESM Figure 5: Venn diagram plots (VDP) summarizing the number of significant metabolites from targeted measurements, in each group from the logistic regression full models. VDP (a) displays the results with all participants and the lower two Venn diagrams show the results with female (b) and male participants (c)

respectively. The number of female participants in each glycemic group: NGR: 173, IGR: 344, T2D: 365; Male participants: NGR: 519, IGR: 1074, T2D: 525. The full statistics are shown in ESM Table 9-14.

ESM Figure 6: Metabolites associated with prediabetes and diabetes from untargeted metabolomics measurements.

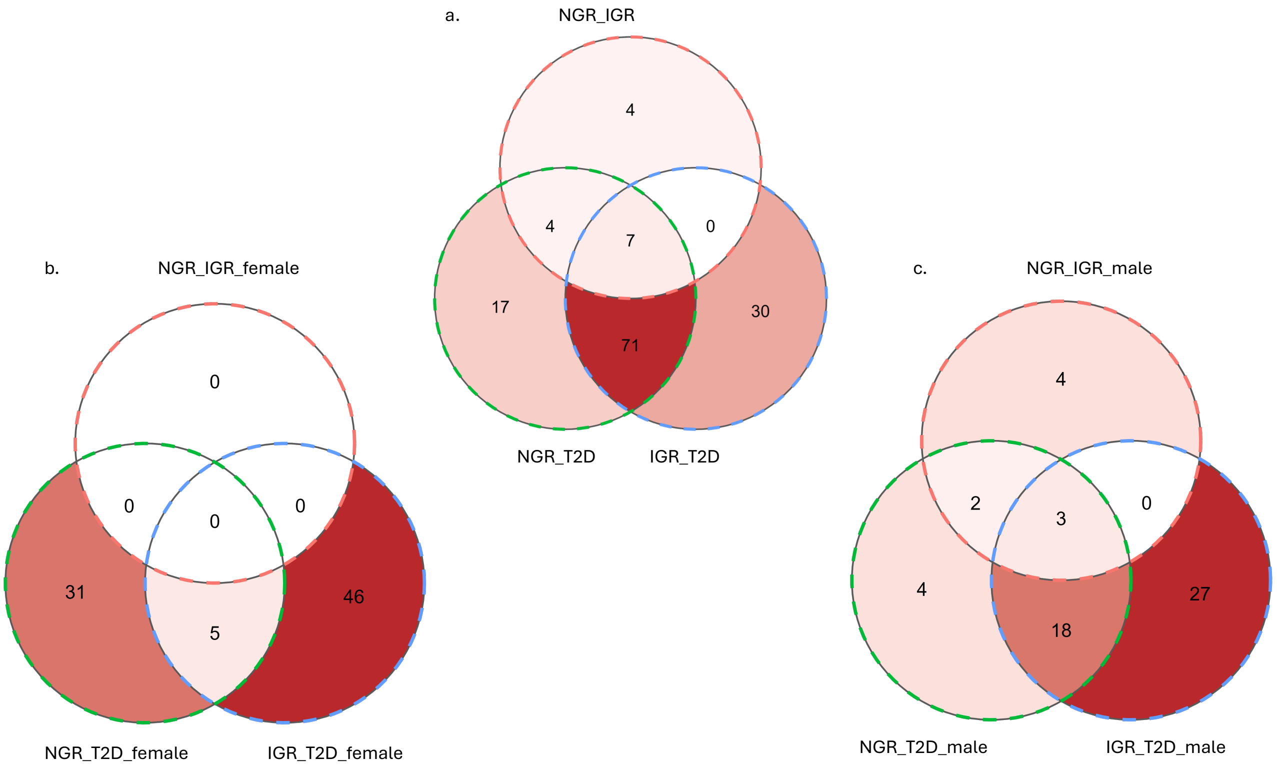

ESM Figure 6: Venn diagram plots (VDP) summarize the number of significant untargeted metabolites in each group from the logistic regression full models. VDP (a) displays the results with all participants and the lower two show the results with female (b) and male participants (c) respectively. The number of female participants in each glycemic group: NGR: 173, IGR: 344, T2D: 365; Male participants: NGR: 519, IGR: 1074, T2D: 525. The full statistics are reported in ESM Table S21-S26.
